# Associations between cardiorespiratory fitness and cardiometabolic risk factors in children and adolescents with obesity

**DOI:** 10.1038/s41598-023-34374-7

**Published:** 2023-05-05

**Authors:** Linnea Johansson, Resthie R. Putri, Pernilla Danielsson, Maria Hagströmer, Claude Marcus

**Affiliations:** 1 Department of Clinical Science, Intervention and Technology, Division of Pediatrics, Karolinska Institutet, CLINTEC, Blickagången 6A, Novum, S-141 57 Huddinge, Sweden; 2 Health Professionals Function, Medical Unit Occupational Therapy and Physiotherapy, Karolinska University Hospital, Stockholm, Sweden; 3 Department of Neurobiology, Care Sciences and Society, Division of Physiotherapy, Karolinska Institutet, Stockholm, Sweden; 4 Academic Primary Health Care Centre, Region Stockholm, Stockholm, Sweden

**Keywords:** Cardiology, Endocrinology, Risk factors

## Abstract

It is unclear if associations between cardiorespiratory fitness (CRF) and cardiometabolic risk factors are independent of degree of obesity, in children with obesity. The aim of this cross-sectional study on 151 children (36.4% girls), 9–17 years, from a Swedish obesity clinic, was to investigate associations between CRF and cardiometabolic risk factors, adjusted for body mass index standard deviation score (BMI SDS), in children with obesity. CRF was objectively assessed with the Åstrand-Rhyming submaximal cycle ergometer test, and blood samples (n = 96) and blood pressure (BP) (n = 84) according to clinical routine. Obesity specific reference values for CRF were used to create CRF levels. CRF was inversely associated with high-sensitivity C-reactive protein (hs-CRP), independent of BMI SDS, age, sex, and height. The inverse associations between CRF and diastolic BP did not remain significant when adjusted for BMI SDS. CRF and high-density lipoprotein cholesterol became inversely associated when adjusted for BMI SDS. Independent of degree of obesity, lower CRF is associated with higher levels of hs-CRP, as a biomarker of inflammation, in children with obesity and regular assessment of CRF should be encouraged. Future research in children with obesity should investigate if low-grade inflammation decreases when CRF is improved.

## Introduction

In children, obesity is related to an increased risk of all-cause mortality, already in young adulthood^1,2^. Pediatric obesity affects the present cardiometabolic risk e.g., increased blood pressure, blood lipids, and inflammatory markers^3–5^. Cardiorespiratory fitness (CRF) is an important marker of health in children^6^ and low levels of CRF are associated with increased cardiometabolic risk factors^7–10^. Adiposity, in terms of body mass index (BMI), waist circumference, and body fat percentage, seems to have a greater impact on cardiometabolic risk in contrast to CRF^11–13^ but results are inconsistent regarding if CRF is independently associated with cardiometabolic risk when adjusted for adiposity^10–12,14^. However, some studies suggest that a high CRF is associated with lower cardiometabolic risk, foremost in children with obesity^8,13^.

Having a CRF equal to or below the lowest quartile or quintile of a reference population is commonly used as a cut-off for low CRF^15,16^. Most children with obesity have a low CRF relative to body weight and significantly lower CRF compared with a normal pediatric population^17^, therefore, the classification of high and low CRF based on a normal population is of limited discriminating clinical value. To facilitate the clinical interpretation o CRF among children with obesity our research group has published age- and sex specific reference values for CRF in this group of children^18^. The reference values are based on CRF assessments, according to the Åstrand-Rhyming submaximal cycle ergometer test^19^, in 705 Swedish children with obesity and CRF percentiles are presented for both absolute maximal oxygen uptake (VO_2max,_ L/min), and for VO_2max_ relative to body weight (mL/kg/min)^18^.

To the best of our knowledge, it has not yet been explored if associations between cardiometabolic risk factors and CRF are present when studying solely children with obesity and adjusting for degree of obesity, in terms of BMI standard deviation score (SDS). Further it remains to be established to which extent different levels of the obesity specific reference values for CRF, developed for clinical practice, are associated with cardiometabolic risk factors. Therefore, the aim of the study was to investigate potential associations between CRF, i.e., absolute- and relative VO_2max_, and cardiometabolic risk factors in children with obesity, and to evaluate how potential associations were affected when adjusted for BMI SDS.

## Methods

### Study design

This was a cross-sectional study of a cohort of children with obesity, starting treatment at Martina Children’s Hospital, in Stockholm, Sweden. The pediatric obesity clinic was established in August 2018 and assessment of CRF commenced in January 2019. Treatment initiation was preceded by clinical evaluation by staff in the obesity team. According to Swedish regulations, guardians and children received verbal and written information about data collection in the Swedish Childhood Obesity Treatment Register (BORIS)^20^, and this study was based on data from BORIS. Informed opt-out consent was obtained from legal guardians, i.e., if the legal guardians did not disapprove, data on height, weight, CRF, blood pressure (BP), and blood samples were registered in BORIS by administrative or clinical staff. The study was approved by the Regional Ethical Committee in Stockholm, Sweden no. 2018/1413-31 and all methods were performed in accordance with relevant guidelines and regulations.

### Participants

Eligible participants were 9–17 years old and had their first appointment between January 2019 and August 2021. Additional inclusion criteria were having obesity according to the International Obesity Task Force (IOTF)^21^, completing a submaximal cycle ergometer test^19^ with a heart rate (HR) ≥ 120 beats per minute (bpm) at the end of the test, and having collected blood samples or BP within 45 days of the performed cycle ergometer test. Children with genetic syndromes, diabetes, heart- or blood disorders or with diagnosed thyroid disease were excluded. Further, children using central nervous system stimulants, long-acting beta2-agonists, or short-acting beta2-agonists (the latter the same day as performing the cycle ergometer test) were not eligible for inclusion. Since this was an explorative study no sample size calculation was conducted.

### Measures and outcomes

At the first appointment to a physiotherapist, anthropometric measures and CRF were assessed. Height was measured without shoes to the nearest 0.1 cm using a stadiometer (Hyssna, Sweden) and weight was measured in light clothing to the nearest 0.1 kg (Seca 899, Germany). Body mass index (BMI) was calculated as weight (kg) divided by height (m) squared (kg/m^2^). Calculation of BMI standard deviation score (SDS) was performed and categorization of degree of obesity (obesity and severe obesity) was based on the IOTF criteria^21^.

CRF, in terms of absolute and relative VO_2max_, was assessed by the Åstrand-Rhyming submaximal cycle ergometer (Monark 874E, Sweden) test^19^. HR was registered every minute by a chest-worn HR monitor (Polar T31, Sweden). Absolute VO_2max_ (L/min) was estimated from workload and the mean HR. If HR differed more than five bpm between minute five and six, the test was prolonged until a constant level was reached. Since oxygen uptake differs in boys and girls, and maximal HR varies with age, absolute VO_2max_ was adjusted for sex and age^19^, with a factor of 1.1 for participants 15 years and younger^22^, and according to Andersson^23^ for older participants. Relative VO_2max_ (mL/kg/min) was calculated by dividing absolute VO_2max_ (converted to mL/min) with weight. The Åstrand-Rhyming nomogram does not enable estimation of VO_2max_ if the mean HR exceeds 140 or 148 bpm (boys and girls respectively) on the minimum workload of 50W/300 kpm, or > 170 bpm for higher workloads (both sexes).

At the first appointment to a pediatrician, BP was measured with an automatic BP monitor (Omron M2, Japan). National guidelines on measuring BP are provided by the Swedish Pediatric Society. A sex-, age-, and height- adjusted reference^24^ was applied when calculating standard deviations scores (SDS) for systolic- and diastolic BP (SBP and DBP). The same reference was used for calculating hypertension, defined as having either a DBP or SBP above the 95^th^ percentile. Most children with obesity ≥ 9 years old were advised to provide blood samples before the first appointment to the pediatrician. Blood samples were collected at the participants’ local health care centers after nightly fast, and all blood sample analyses were conducted by Swedish laboratories with official authorization. Blood samples included parameters following the clinical routine i.e., alanine aminotransferase (ALT), fasting glucose, fasting insulin, glycated hemoglobin A1c (HbA1c), low-density lipoprotein (LDL) cholesterol, high-density lipoprotein (HDL) cholesterol, total cholesterol, triglycerides, and high-sensitivity C-reactive protein (hs-CRP). Homeostasis model assessment of insulin resistance (HOMA-IR) was calculated as (fasting glucose [mmol/L] x fasting insulin [µU/L])/22.5.

Elevated ALT was defined as > 0.37 µkat/L in girls and > 0.44 µkat/L in boys^25^. Lipid profile was categorized as having low HDL cholesterol (< 1.0 mmol/L), elevated LDL cholesterol (≥ 2.8 mmol/L), elevated total cholesterol (≥ 4.4 mmol/L), and elevated triglycerides (≥ 0.8 mmol/L for 9-year-old participants and ≥ 1.0 mmol/L in participants ≥ 10 years)^26^. Low-grade inflammation (LGI) was defined as a hs-CRP > 3 to ≤ 10 mg/L^27^. A CRP > 10 mg/L was considered as an ongoing high-grade inflammation^28^ and therefore excluded from analyses (n = 1). Impaired fasting glucose (IFG) was defined as a fasting glucose ≥ 6.1 mmol/L^29^.

### Statistical analysis

Continuous data are presented with mean and standard deviation (SD) or with median and interquartile range (IQR) based on data distribution. Categorical variables are presented with frequency and/or percentage. Comparisons of two groups (included vs. subgroups of excluded children; valid estimation of VO_2max_ vs. HR too high for estimating VO_2max_) were analyzed with the Student’s t-test, the Mann–Whitney U test, or the Chi-square test. For all other analyses, solely participants with a valid estimation of VO_2max_ were studied.

To investigate associations between cardiometabolic risk and CRF (i.e., absolute- and relative VO_2max_), linear regressions were conducted with cardiometabolic risk factors as dependent variables, and absolute VO_2max_ (L/min) or relative VO_2max_ (mL/kg/min) as independent variables. CRF was adjusted for age, sex, and height (Model 1) as well as BMI SDS, age, sex, and height (Model 2). HOMA-IR, fasting insulin, hs-CRP, ALT, HDL, and triglycerides were logarithmically transformed to meet the assumptions of linear regression. For linear regressions the unstandardized beta is presented.

Participants were categorized into three CRF levels based on reference values for children with obesity, according to sex and age group^18^. Differences of biochemical markers and BP between the three groups were analyzed with one-way analysis of variance (ANOVA) with the Tukey’s post-hoc test, or the Kruskal Wallis test with the Bonferroni post-hoc test. To compare proportions of the outcome variables between the three groups, Chi-square test was applied. If significant, pairwise comparisons of predictive margins, adjusted with the Bonferroni method were further conducted to identify the difference between two groups. One-way analysis of covariance (ANCOVA), with Bonferroni correction, was used to compare differences in logarithmically transformed hs-CRP between the three CRF groups, adjusted for BMI SDS. All p-values for comparing proportion, mean, and rank (Tables 2 and 3) were adjusted for multiple testing by multiplying the uncorrected p-values with number of tests. A *p*-value of < 0.05 was considered as statistically significant. IBM SPSS version 28 (IBM SPSS Armonk, NY, USA) was used for all calculations except for pairwise comparisons of predictive margins where STATA version 16 (StataCorp, College Station, TX, USA) was used.

## Results

**Figure 1:**
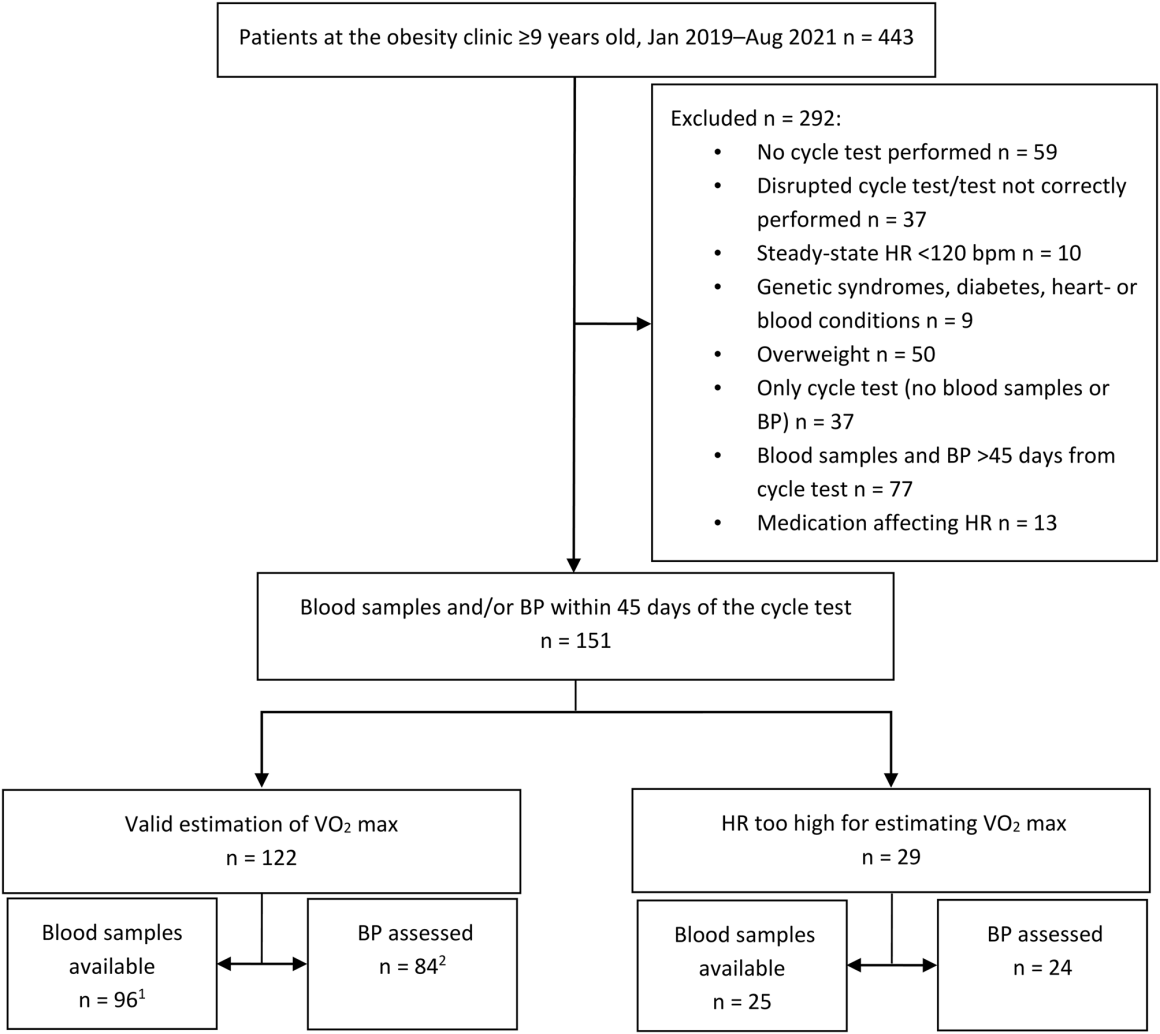
Flowchart for identification of inclusion and exclusion in the study. ^1^Final sample for the association between CRF and biomarkers, ^2^Final sample for the association between CRF and BP. Abbreviations: HR, heart rate; BP, blood pressure; VO_2_ max, maximal oxygen uptake.

A flowchart for identification of inclusion and exclusion is presented in Fig. 1. Of the 151 included subjects, with a mean age of 13.1 (SD 1.9) years, 36.4% were girls. Of the participants with data on migrant background (86%), 61% had one or two parents born outside Scandinavia, and 47% came from countries outside Europe. Asthma was present in 15 subjects (10%) and 14 participants (9%) were diagnosed with attention deficit hyperactivity disorder or attention deficit disorder without ongoing pharmaceutical treatment. A valid estimation of VO_2max_ was present in 81% (n = 122) of the subjects. of which blood samples were taken in 96 individuals and BP in 84 subjects. Of these participants, 58 children had available data on both blood samples and BP and the other children are unique for each group (blood samples vs BP, Fig. 1). The median (IQR) time between the cycle ergometer test and blood samples were 0 (20) days, and 0 (32) days for measured BP.

Linear regressions (Table 1) showed that Log_10_ hs-CRP was inversely associated with relative VO_2max_ (β = − 0.022, *p* = 0.013) and absolute VO_2max_ (β = − 0.238, *p* = 0.015) when adjusted for BMI SDS (Model 2). In other words, an increment in relative VO_2max_ by 1 mL/kg/min is associated with a 4.9% lower hs-CRP, and for absolute VO_2max_ an increase by 1 L/min is associated with a 42.2% lower hs-CRP value. Model 2 showed inverse associations between Log_10_ HDL and relative VO_2max_ (β = − 0.005, *p* = 0.005), and absolute VO_2max_ (β = − 0.053, *p* = 0.026), but no significant associations were found in Model 1. Log_10_ fasting insulin, Log_10_ HOMA-IR, Log_10_ ALT, fasting glucose, HbA1c, Log_10_ triglycerides, LDL-, and total cholesterol were not significantly associated with CRF in any of the adjustment models. Of participants with data on BP (n = 84), a significant inverse association between relative VO_2max_ and DBP SDS in Model 1 did not remain significant in Model 2. No significant associations could be detected between CRF and SBP SDS.

**Table 1:** Associations of cardiorespiratory fitness and cardiometabolic risk factors.

Dependent variable	Model 1 Relative VO_2max_ (mL/kg/min)			Model 2 Relative VO_2max_ (mL/kg/min)		
	Beta coefficient^1^	95% CI	*P*	Beta coefficient^1^	95% CI	*P*
Log_10_ hs-CRP^a^	− .027	− .042 to − .012	** < .001**	− .022	− .039 to − .005	**.013**
Log_10_ HDL^c^	− .002	− .006 to .002	.213	− .005	− .009 to − .002	**.005**
Log_10_ fasting insulin^b^	− .008	− .016 to − .001	.080	.001	− .008 to .010	.834
Log_10_ HOMA-IR^c^	− .008	− .017 to − .001	.085	.002	− .008 to .011	.747
Log_10_ ALT^d^	− .002	− .012 to .008	.661	.001	− .009 to .0.12	.792
Fasting glucose^c^	− .005	− .019 to .009	.446	.005	− .010 to .020	.533
HbA1c^b^	− .073	− .189 to .044	.221	.023	− .104 to .149	.724
Log_10_ triglycerides^c^	− .006	− .013 to .001	.102	− .005	− .013 to .004	.276
LDL^c^	− .003	− .031 to .025	.844	− .009	− .041 to .023	.589
Total cholesterol^c^	− .011	− .037 to .016	.424	− .023	− .053 to .007	.137
DBP SDS^e^	− .032	− .063 to -.001	**.042**	− .010	− .046 to .025	.566
SBP SDS^e^	− .025	− .061 to .011	.166	− .012	− .054 to .031	.586

	Model 1Absolute VO_2max_ (L/min)			Model 2 Absolute VO_2max_ (L/min)		
Log_10_ hs-CRP^a^	− .262	− .486 to − .038	**.024**	− .238	− .457 to − .019	**.015**
Log_10_ HDL^c^	− .047	− .096 to .001	.055	− .053	− .101 to − .006	**.026**
Log_10_ fasting insulin^b^	− .014	− .137 to .110	.825	.010	− .103 to .123	.864
Log_10_ HOMA-IR^c^	− .011	− .142 to .120	.870	.015	− .104 to .134	.799
Log_10_ ALT^d^	− .163	− .910 to .584	.666	− .125	− .870 to .620	.740
Fasting glucose^c^	.012	− .184 to .209	.901	.038	− .152 to .228	.690
HbA1c^b^	.073	− 1.596 to 1.743	.931	.328	− 1.258 to 1.914	.682
Log_10_ triglycerides^c^	− .061	− .164 to .043	.246	− .054	− .158 to .049	.301
LDL^c^	− .019	− .418 to .379	.924	− .031	− .433 to .371	.879
Total cholesterol^c^	− .153	− .531 to .226	.425	− .173	− .553 to .206	.366
DBP SDS^e^	− .183	− .622 to .257	.410	− .121	− .541 to .300	.570
SBP SDS^e^	− .178	− .681 to .326	.484	− .136	− .636 to .364	.589

VO_2max_, maximal oxygen uptake; hs-CRP, high-sensitive C-reactive protein; HOMA-IR, homeostasis model assessment of insulin resistance; HDL, high-density lipoprotein; ALT, alanine aminotransferase; HbA1c, glycated hemoglobin A1c; LDL, low-density lipoprotein; DBP SDS, diastolic blood pressure standard deviation score; SBP SDS, systolic blood pressure standard deviation score; CI, confidence interval. Model 1, CRF adjusted for age (years), sex, and height (m); Model 2, CRF adjusted for BMI SDS, age (years), sex, and height (m). ^1^Unstandardized beta. ^a^n = 92; ^b^n = 96; ^c^n = 95; ^d^n = 94; ^e^n = 84.* P*-values < .05 was considered as statistically significant (marked with bold).

Group comparisons between CRF levels of relative VO_2max_ (mL/kg/min) are presented in Tables 2, 3. Hs-CRP increased for each group with lower CRF, where the group with lowest CRF had a median (IQR) of 4.0 (3.0) mg/L vs 1.5 (2.3) mg/L in the group with highest CRF (*p* = 0.021).

**Table 2:** Group comparisons between levels of relative VO_2max_ (mL/kg/min) in participants with available blood samples (n = 96).

Variables	≤ 25th percentile	n	> 25th to ≤ 50th percentile	n	> 50th percentile	n	*P* Value^1^
Girls, n (%)	8 (27.6)		10 (45.5)		15 (33.3)		1.00
Age (years), mean (SD) [min–max]	13.6 (1.6) [10.3–16.1]	29	13.6 (2.0) [10.6–17.1]	22	13.4 (1.8) [9.4–16.6]	45	1.00
Migrant background, n (%)^2^	10 (41.7)	24	12 (57.1)	21	15 (41.7)	36	1.00
BMI SDS, mean (SD) [min–max]	2.98 (0.28) [2.35–3.43]^a,b^	29	2.77 (0.28) [2.33–3.46]^a^	22	2.63 (0.26) [2.19–3.11]^b^	45	**.021**
Severe obesity, n (%)	17 (58.6)^b^	29	6 (27.3)	22	8 (17.8)^b^	45	**.021**
hs-CRP (mg/L), median (IQR) [min–max]	4.0 (3.0) [0.7–9.9]^b^	27	2.5 (4.3) [0.2–9.9]	20	1.5 (2.3) [0.2–10.0]^b^	45	**.021**
LGI, n (%)	17 (63.0)^b^		8 (40.0)		10 (22.2)		.063
ALT (µkat/L), median (IQR) [min–max]	0.42 (0.49) [0.17–2.64]	28	0.37 (0.43) [0.21–2.31]	22	0.42 (0.34) [0.16–2.93]	44	1.00
Elevated ALT, n (%)	13 (46.4)		9 (40.9)		21 (47.7)		1.00
HDL (mmol/L), median (IQR) [min–max]	1.1 (0.3) [0.7–1.9]	29	1.0 (0.5) [0.7–1.7]	21	1.2 (0.3) [0.7–1.6]	45	1.00
Low HDL, n (%)	6 (20.7)		9 (42.9)		7 (15.6)		.966
LDL (mmol/L), mean (SD) [min–max]	2.5 (0.7) [1.0–4.3]	29	2.6 (0.9) [1.3–4.4]	21	2.5 (0.8) [1.1–4.9]	45	1.00
Elevated LDL, n (%)	7 (24.1)		8 (38.1)		14 (31.1)		1.00
Total cholesterol (mmol/L), mean (SD) [min–max]	4.0 (0.6) [2.7–5.5]	29	4.1 (0.8) [3.0–5.6]	21	4.1 (0.8) [2.1–5.9]	45	1.00
Elevated total cholesterol, n (%)	9 (31.0)		8 (38.1)		14 (31.1)		1.00
Triglycerides (mmol/L), median (IQR) [min–max]	1.2 (0.9) [0.5–2.8]	29	0.9 (0.5) [0.5–2.3]	21	0.9 (0.6) [0.4–3.4]	45	1.00
Elevated triglycerides, n (%)	17 (58.6)		9 (42.9)		21 (46.7)		1.00
Glucose (mmol/L), mean (SD) [min–max]	5.5 (0.3) [5.0–6.1]	29	5.5 (0.4) [4.9–6.1]	21	5.4 (0.4) [4.1–6.3]	45	1.00
IFG, n (%)	1 (3.4)		3 (14.3)		2 (4.4)		NA
Insulin (µU/L), median (IQR) [min–max]	24 (22) [11–98]	29	22 (19) [6–99]	22	21 (11) [9–54]	45	1.00
HOMA-IR, median (IQR) [min–max]	6.24 (6.35) [2.44–24.83]	29	4.99 (5.44) [1.29–25.48]	21	4.80 (2.55) [2.04–14.16]	45	1.00
HbA1c, mean (SD) [min–max]	36 (3) [31–44]	29	35 (3) [30–41]	22	35 (3) [27–41]	45	1.00

ALT, alanine aminotransferase; HDL, high-density lipoprotein; LDL, low-density lipoprotein; IFG, impaired fasting glucose; HOMA-IR, homeostasis model assessment of insulin resistance; HbA1c, glycated hemoglobin A1c; hs-CRP, high-sensitive C-reactive protein; LGI, low-grade inflammation; BMI SDS, body mass index standard deviation score. Percentile groups are based on reference values for cardiorespiratory fitness in children with obesity (Johansson et al.^18^). *P*-values were derived from one-way ANOVA, the Kruskal Wallis test, or the Chi square test, where appropriate, and were adjusted for correction for multiple testing (uncorrected *p*-value × 21 tests). NA indicates that groups were too small for comparison. *P*-values < .05 was considered as statistically significant (marked with bold). ^1^*P-*values for differences between the three groups are shown in the table. If significant, post-hoc analysis was performed adjusted with the Bonferroni or Tukey’s method, ^2^One or two parents born outside Europe, ^a^ Significant difference between the groups of ≤ *25th percentile* and > *25th to* ≤ *50th percentile*; ^b^ Significant difference between the groups of ≤ *25th percentile* and > *50th percentile.*

**Table 3:** Group comparisons between levels of relative VO_2max_ (mL/kg/min) in participants with measured blood pressure (n = 84).

Variables	≤ 25th percentile, n = 30	> 25th to ≤ 50th percentile, n = 22	> 50th percentile, n = 32	*P* Value^1^
Girls, n (%)	9 (30.0)	12 (54.5)	10 (31.3)	1.00
Age (years), mean (SD) [min–max]	13.7 (1.5) [10.5–16.7]	13.8 (1.9) [10.2–17.1	13.5 (1.5) [9.9–16.0]	1.00
Migrant background, n (%)^2^	12 (44.4)^3^ n = 27	14 (66.7)^5^	9 (39.1)^5^	1.00
BMI SDS, mean (SD) [min–max]	3.10 (0.27) [2.42–3.54]^a,b^	2.86 (0.30) [2.33–3.46]^a,c^	2.64 (0.26) [2.19–3.11]^b,c^	**.007**
Severe obesity, n (%)	23 (76.7)^b^	10 (45.5)	6 (18.8)^b^	**.007**
SBP SDS, mean (SD) [min–max]	0.32 (0.79) [− 1.29–1.89]	0.28 (1.06) [− 1.66–2.55]	− 0.22 (0.85) [− 2.11–1.29]	.252
DBP SDS, mean (SD) [min–max]	1.09 (0.91) [− 0.47–3.00]	0.96 (0.80) [− 0.33—3.44]	0.67 (0.58) [− 0.54–2.11]	.651
Hypertension, n (%)	10 (33.3)	4 (18.2)	2 (6.3)	NA

SBP SDS, systolic blood pressure standard deviation score; DBP SDS, diastolic blood pressure standard deviation score; BMI SDS, body mass index standard deviation score. Percentile groups are based on reference values for cardiorespiratory fitness in children with obesity (Johansson et al.^18^). *P*-values, adjusted for correction for multiple testing (uncorrected *p*-value × 7 tests), derive from one-way ANOVA, the Kruskal Wallis test, or the Chi square test, where appropriate. NA indicates that groups were too small for comparison. *P*-values < .05 was considered as statistically significant (marked with bold). ^1^*P-*values for differences between the three groups are shown in the table. If significant, post-hoc analysis was performed adjusted with the Bonferroni or Tukey’s method. ^2^One or two parents born outside Europe. ^3^n = 27, ^4^ n = 21, ^5^ n = 23, ^a^Significant difference between the groups of ≤ *25th percentile* and > *25th to* ≤ *50th percentile*; ^b^Significant difference between the groups of ≤ *25th percentile* and > *50th percentile*; ^c^Significant difference between the groups of > *25th to* ≤ *50th percentile* and > *50th percentile.*

The one-way ANCOVA (Levene’s test *p* = 0.04) showed that the significant difference of Log_10_ hs-CRP between the lowest and the highest CRF levels remained when adjusted for BMI SDS (*p* = 0.026). The same trend was seen in LGI, but group differences were non-significant (*p* = 0.063). The lower CRF-level, the higher proportion of severe obesity and mean BMI SDS were found. For the other biomarkers, BP, or descriptive variables no group differences could be detected (Tables 2, 3). For absolute VO_2max_ (L/min), no significant group differences were found for biochemical markers, BP, age, sex, or BMI SDS.

Data on study participants and subgroups of excluded subjects are presented in Table 4. The excluded group without available data on blood samples and BP (n = 37) were younger (*p* = 0.017) and had lower BMI SDS (*p* < 0.001) compared with included subjects (n = 151). The excluded subgroup with blood samples and BP assessed more than 45 days from the cycle ergometer test (n = 77) had significantly fewer individuals with a HR too high for estimating VO_2max_ (*p* = 0.024). The included subjects had a significantly lower mean value of relative VO_2max_ (mL/kg/min) compared with the other groups (*p* = 0.044 and *p* = 0.026).

**Table 4:** Comparison of included- and subgroups of excluded subjects.

Variables	Included	Excluded^a^	Excluded^b^	*P* value^1^	*P* value^2^
	n = 151	n = 37	n = 77		
Age (years), mean (SD)	13.1 (1.9)	12.2 (2.2)	12.8 (2.1)	**.017**	.256
Girls, n (%)	55 (36.4)	15 (40.5)	31 (40.3)	.642	.572
BMI SDS, mean (SD)	2.80 (0.33)	2.57 (0.25)	2.74 (0.35)	** < .001**	.196
Severe obesity, n (%)	54 (35.8)	3 (8.1)	24 (31.2)	** < .001**	.489
VO_2max_ (L/min), median (IQR)	2.2 (0.5)^c^	2.0 (0.7)^d^	2.2 (0.4)^e^	.279	.898
VO_2max_ (mL/kg/min), mean (SD)	27.0 (6.3)^c^	29.7 (6.0)^d^	29.1 (5.6)^e^	**.044**	**.026**
HR too high for VO_2_ max estimation, n (%)	29 (19.2)	10 (27.0)	6 (7.8)	.293	**.024**
Date (month, year) for cycle test, min–max	Feb 2019	Apr 2019	Feb 2019		
	Apr 2021	Jun 2021	Apr 2021		

BP, blood pressure; BMI SDS, body mass index standard deviation score; VO_2max_, maximal oxygen uptake; HR, heart rate. *Excluded*^*a*^*;* bloods samples and BP not assessed. *Excluded*^*b*^*;* Blood samples and BP assessed > 45 days from the cycle ergometer test. *P*-values derive from the Chi-square test, Student’s t-test, or Mann–Whitney U test, where appropriate. *P*-values < .05 was considered as statistically significant (marked with bold).^1^Comparison between *Included* and *Excluded*^*a*^; ^2^Comparison between *Included* and *Excluded*^*b* a^Cycle ergometer test performed but no available blood samples or BP; ^b^Blood samples and BP > 45 days from performed cycle ergometer test; ^c^Valid estimation of VO_2_ max n = 122; ^d^Valid estimation of VO_2_ max n = 27; ^e^Valid estimation of VO_2_ max n = 71.

For 29 (19%) of the included participants, HR during the cycle ergometer test was too high to estimate VO_2max_ with the Åstrand-Rhyming nomogram. These individuals were significantly younger compared to other participants (mean (SD) age of 11.6 (1.7) vs. 13.5 (1.8), *p* < 0.001) and within the group, 22 (76%) individuals were younger than 12 years old. Further, the group with high HR had higher HDL cholesterol levels with a median (IQR) of 1.3 (0.3) vs. 1.2 (0.3), *p* = 0.005. No other significant group differences could be detected.

## Discussion

In this cross-sectional study of children and adolescents with obesity, we found an inverse association between cardiorespiratory fitness and hs-CRP, that was independent of BMI SDS. CRF (mL/kg/min) equal to or below the 25th percentile, according to reference values in children with obesity^18^, entailed significantly higher levels of inflammation, in terms of increased hs-CRP. These results add to existing literature by investigating the influence of CRF on hs-CRP within children with obesity, including the continuous variable of BMI SDS as an adjustment for the impact of obesity.

Elevated inflammatory markers are associated with obesity-related disease in children^5,30^. LGI in individuals with obesity is involved in the pathogenesis of multiple severe health conditions such as autoimmune diseases, atherosclerosis, and cardiovascular disease^31–33^. Exercise training has been found to reduce hs-CRP in adults, independent of BMI reduction^34^. The mechanisms behind the effects of exercise training on LGI are not fully understood, however, several pathways are suggested to be involved, including muscle- and adipose tissue as well as endothelial- and immune cells^35^. In children, the current literature is mixed regarding if hs-CRP is associated with CRF when adjusted for adiposity^10,36–39^, however, previous studies mainly included children with normal weight. Since children with obesity have significantly higher prevalence of low-grade inflammation compared to children with normal weight^5^ it is important to study children with obesity separately. In a pooled cross-sectional analysis of 1 706 adolescents, Agostinis-Sobrinho et al.^40^ found that high CRF attenuated the association between hs-CRP and BMI, mainly for the adolescents with obesity. Similar findings, where high CRF was associated with a lower cardiometabolic risk score solely in children with obesity, have been presented by Nyström et al.^8^. This indicates that CRF may be important for decreasing cardiometabolic risk, especially in children with obesity.

In contrast to other studies^41,42^ we found no associations between VO_2max_ (mL/kg/min) and SBP SDS, ALT, fasting insulin, HOMA-IR, fasting glucose, HbA1c, triglycerides, and LDL-, or total cholesterol. This may partly be explained by the relatively small study population. Further, the inclusion of solely children with obesity may have resulted in a narrower distribution of cardiometabolic risk outcomes compared with including children of different weight status. HDL cholesterol was inversely associated with CRF, but only when adjusted for BMI SDS. Further, when CRF was categorized no differences in HDL distribution could be detected between the different CRF levels. Additional studies with larger study populations should be conducted to either confirm or reject our findings.

Similarly to hs-CRP and CRF, the literature is mixed regarding if CRF is independently associated with other measures of cardiometabolic risk in children^10–12,14^. The diverse findings in the current literature may be explained by how adiposity and CRF is measured^43^. The widely used measurement of BMI and VO_2max_ relative to body weight (assessed by indirect exercise tests), have been criticized for being imprecise measures of adiposity and CRF^44^. Fat free mass (FFM) is independent of adiposity and highly influential on VO_2max_ in children and therefore a better indicator of physiological ability to maximally consume oxygen^44,45^. However, findings also differed when VO_2max_ relative to FFM was used in adolescents and young adults of different weight status, from being significantly associated with^46^ or not associated with cardiometabolic risk factors^43^. However, when solely children with obesity were studied, VO_2max_ relative to FFM was positively associated with insulin sensitivity^9^. Precise measures of adiposity, FFM (e.g., by dual-energy X-ray absorptiometry) and CRF (i.e., direct VO_2max_ tests including analysis of gas exchange) are expensive and therefore not applicable in population-based or clinical settings. Moreover, direct VO_2max_ assessment is highly dependent on motivation to get a valid test result and therefore difficult to use in untrained children. Nevertheless, assessing CRF among children in obesity treatment serves many purposes—from assessing functional ability to evaluating effects from exercise prescription. Moreover, since CRF is an important marker for health in children, clinically available assessments of CRF and adiposity are essential. However, since our findings were based on VO_2max_ relative to body weight and adjusted for BMI SDS, the inverse relationship between hs-CRP and CRF may not be fully independent of adiposity.

No significant group differences were found between absolute VO_2max_ and BMI SDS, BP, or any of the analyzed biochemical markers. Since absolute VO_2max_ in children with obesity are similar, or higher than values from a general population^17,45^, these findings were not surprising. Although absolute VO_2max_ should be reported to understand changes in CRF, VO_2max_ relative to body weight or FFM serves a better purpose when comparing differences between individuals^45,47^.

Subjects included in this study differed significantly from excluded subgroups. Individuals without available blood samples or BP were younger and had a lower BMI SDS. This suggests that the clinical evaluation resulted in that blood samples for the identification of co-morbidities were not indicated. Because of the COVID-19 pandemic, appointments to the physiotherapist and pediatrician were further apart than intended, which may be a reason to why 77 children had BP and blood samples taken more 45 days from the performed cycle test. The studied population had lower relative VO_2max_ (mL/kg/min) than the excluded subgroups; hence, the detected associations between CRF and cardiometabolic risk may have been stronger if patients at the obesity clinic with higher CRF levels could have been included.

The Åstrand-Rhyming cycle ergometer test was not developed for children^19^ and the test has not been validated for children with obesity. In children with normal weight the Åstrand-Rhyming test has similar validity compared with other indirect tests for CRF^48^. As previously stated in this discussion, indirect tests have several advantages and for children with obesity cycle tests enable assessment of CRF in those experiencing pain when walking or running. In our study, participants with a HR too high to estimate VO_2max_ were significantly younger than the other subjects, and 76% of these children were younger than 12 years old. This indicates that the Åstrand-Rhyming test is not optimal for the youngest children, which is in accordance with previous findings^18^. Nevertheless, using the test in younger children may still be of clinical importance to assess level of exertion, changes in HR and ability to maintain speed. For children 12 years and older, a HR too high to estimate VO_2max_ may indicate that CRF levels are extremely low.

Several limitations to this study have previously been stated in the discussion. Additionally, this study is limited using clinical data resulting in a time difference between assessed BP together with blood samples and CRF. However, weight changes during the BP and blood sample collection, if any, is believed to be modest. Further, our results are in line with previous findings that CRF attenuate the effects of obesity on cardiometabolic risk in children^8,9,13,40^. In accordance with many studies reporting fitness outcomes in children^11,49,50^, this study is limited by not having available data on pubertal stage—a variable related to CRF in children^44^.We strived to eliminate other confounders for CRF and cardiometabolic risk factors by excluding children with diseases or medications affecting HR, BP, or biochemical markers. The inclusion of solely children with obesity, of different migrant background, was a strength of this study. That our findings are presented in relation to reference values for CRF in children with obesity, enable interpretation of CRF assessed in a clinical setting. This will allow further investigation regarding if also minor improvements of CRF in children with obesity could decrease hs-CRP.

### Clinical implications

Our results indicate that there is an association between lower CRF and increased hs-CRP in children with obesity. Low-grade inflammation in young children seems to be an important factor behind the association between pediatric obesity and autoimmune disease and cancers later in life^32,51^. Inflammation is also involved in the development of atherosclerosis and type 2 diabetes^31,33,51^. Thus, it is of major importance to reduce inflammation in young children with obesity. Exercise training in adults can reduce inflammation^34^ and it is likely, but not yet demonstrated, that improvements of CRF according to the reference values in children^18^, would reduce inflammatory markers. Previous findings indicate that high-intensity interval training may improve CRF more than moderate-intensity continuous training, in children with obesity^49^. However, for children with obesity, increased physical activity can be challenging—especially high-intensity exercise. Further, it is likely a high individual variation to which extent physical activity will improve CRF. Therefore, CRF and markers for low-grade inflammation should be assessed regularly in children with obesity. The reference values of CRF in this population^18^ could be helpful for initial clinical evaluation to obtain an understanding of the patient’s CRF health status.

## Conclusions

When using recently developed reference values for CRF, which enable improved grading of CRF for children with obesity, we found that children with lower CRF had more pronounced signs of inflammation in terms of increased hs-CRP, regardless of degree of obesity. As low-grade inflammation early in life is of major importance for later development of obesity related comorbidities, regular assessment of CRF and exercise interventions to increase CRF should be encouraged. Future research, including exercise interventions in children with obesity, are needed to investigate to which extent low-grade inflammation decreases when CRF is improved.

## Data Availability

The datasets are available from the corresponding author on reasonable request.

## Abbreviations

CRF: Cardiorespiratory fitness
BP: Blood pressure
hs-CRP: High-sensitivity C-reactive protein
HDL: High-density lipoprotein
LDL: Low-density lipoprotein
BMI: Body mass index
SDS: Standard deviation score
VO_2max_: Maximal oxygen uptake
BORIS: Swedish Childhood Obesity Treatment Register
IOTF: International Obesity Task Force
HR: Heart rate; bpm, beats per minute
SBP: Systolic blood pressure
DBP: Diastolic blood pressure
ALT: Alanine aminotransferase
HbA1c: Glycated hemoglobin A1c
HOMA-IR: Homeostasis model assessment of insulin resistance
LGI: Low-grade inflammation
IFG: Impaired fasting glucose
